# Rabies Elimination in Rural Kenya: Need for Improved Availability of Human Vaccines, Awareness and Knowledge on Rabies and Its Management Among Healthcare Workers

**DOI:** 10.3389/fpubh.2022.769898

**Published:** 2022-03-10

**Authors:** Veronicah Mbaire Chuchu, Philip Mwanzia Kitala, Philet Bichanga, Daniel Ksee, Mathew Muturi, Athman Mwatondo, Carolyne Nasimiyu, Marybeth Maritim, Nyamai Mutono, Tariku J. Beyene, Sophie Druelles, Katie Hampson, S. M. Thumbi

**Affiliations:** ^1^Department of Public Health, Pharmacology and Toxicology, University of Nairobi, Nairobi, Kenya; ^2^Center for Global Health Research, Kenya Medical Research Institute, Kisumu, Kenya; ^3^Washington State University Global Health Program, Nairobi, Kenya; ^4^Government of Makueni County, Department of Health Services, Wote, Kenya; ^5^Government of Makueni County, Department of Agriculture, Irrigation, Livestock, and Fisheries Development, Wote, Kenya; ^6^Zoonotic Disease Unit, Ministry of Health and Ministry of Agriculture, Livestock and Fisheries, Nairobi, Kenya; ^7^Department of Clinical Medicine and Therapeutics, University of Nairobi, Nairobi, Kenya; ^8^Center for Epidemiological and Modelling Analysis, Institute of Tropical and Infectious Diseases, University of Nairobi, Nairobi, Kenya; ^9^Center for Health Equity and Outcomes Research, The Research Institute at Nationwide Children's Hospital, Columbus, OH, United States; ^10^Vaccine Epidemiology and Modeling, Sanofi Pasteur, Lyon, France; ^11^Institute of Biodiversity, Animal Health & Comparative Medicine, University of Glasgow, Glasgow, United Kingdom; ^12^Institute of Immunology and Infection Research, School of Biological Sciences, University of Edinburgh, Edinburgh, United Kingdom; ^13^NIHR Global Health Research Unit Tackling Infections to Benefit Africa (TIBA), The University of Edinburgh, Edinburgh, United Kingdom; ^14^Paul G Allen School for Global Health, Washington State University, Pullman, WA, United States

**Keywords:** rabies, awareness, knowledge, post-exposure-prophylaxis, rabies immunoglobulin

## Abstract

**Background:**

In Africa, rabies causes an estimated 24,000 human deaths annually. Mass dog vaccinations coupled with timely post-exposure prophylaxis (PEP) for dog-bite patients are the main interventions to eliminate human rabies deaths. A well-informed healthcare workforce and the availability and accessibility of rabies biologicals at health facilities are critical in reducing rabies deaths. We assessed awareness and knowledge regarding rabies and the management of rabies among healthcare workers, and PEP availability in rural eastern Kenya.

**Methodology:**

We interviewed 73 healthcare workers from 42 healthcare units in 13 wards in Makueni and Kibwezi West sub-counties, Makueni County, Kenya in November 2018. Data on demographics, years of work experience, knowledge of rabies, management of bite and rabies patients, and availability of rabies biologicals were collected and analyzed.

**Results:**

Rabies PEP vaccines were available in only 5 (12%) of 42 health facilities. None of the health facilities had rabies immunoglobulins in stock at the time of the study. PEP was primarily administered intramuscularly, with only 11% (*n* = 8) of the healthcare workers and 17% (7/42) healthcare facilities aware of the dose-sparing intradermal route. Less than a quarter of the healthcare workers were aware of the World Health Organization categorization of bite wounds that guides the use of PEP. Eighteen percent (*n* = 13) of healthcare workers reported they would administer PEP for category I exposures even though PEP is not recommended for this category of exposure. Only one of six respondents with acute encephalitis consultation considered rabies as a differential diagnosis highlighting the low index of suspicion for rabies.

**Conclusion:**

The availability and use of PEP for rabies was sub-optimal. We identified two urgent needs to support rabies elimination programmes: improving availability and access to PEP; and targeted training of the healthcare workers to improve awareness on bite wound management, judicious use of PEP including appropriate risk assessment following bites and the use of the dose-sparing intradermal route in facilities seeing multiple bite patients. Global and domestic funding plan that address these gaps in the human health sector is needed for efficient rabies elimination in Africa.

## Introduction

Rabies is a fatal viral disease causing an estimated 59,000 human deaths every year globally. Most of these deaths occur in the rural populations in Africa and Asia, and among children 15 years of age and below (1, 2). The domestic dog is the primary source of human cases of rabies (3). As such, human rabies is 100% preventable through three complementary interventions. First, mass dog vaccination that achieve 70% herd immunity to break dog-to-dog transmission and reduces the risk of rabies exposure to humans. Second, prompt post-exposure prophylaxis (PEP) for people bitten by suspect rabid dogs. This includes thorough wound washing, administration of rabies post-exposure vaccines, and infiltration of rabies immunoglobulins (RIG) for persons with risky bites (multiple bites, bites in the upper trunk), referred to as category 3 bites as outlined in the WHO protocol (4). Third, raising awareness on rabies in the community and among healthcare workers to improve the delivery of dog vaccinations, prevention of dog bites, health-seeking behavior, and access to appropriate PEP following bites. These interventions form the key strategies for the global goal of ending human deaths from dog-mediated rabies by 2030 (5, 6).

In Kenya, rabies is endemic across the country and has been estimated to cause 523 (95% CI 134, 1,100) deaths annually (2, 7). The country is implementing a strategy to end human deaths from rabies by 2030 following the step-wise progressive reduction in disease burden approach (starting in elimination activities in select pilot counties and progressively extending to the rest of the country) (7). The strategy combines mass dog vaccination, prompt provision of PEP, public awareness on rabies, and enhanced surveillance of the disease in animal and human populations as key activities (7).

Our previous study on the rabies vaccines and immunoglobulin supply and logistics system in Kenya have shown large variability in PEP availability across different regions in the country, and the predominant use of the intramuscular route for administration of PEP vaccines (8). This is despite the World Health Organization (WHO) recommendations for the use of dose-sparing and cost-saving intradermal administration, which allows for more bite patients to receive the life-saving vaccine (9, 10). WHO has provided updated guidelines on rabies pre-exposure and post-exposure vaccination strategies with recommendations for countries to switch to intradermal injections; to undertake risk assessments to determine if PEP or RIG is required, and to discontinue PEP if the biting animal is confirmed to be negative for rabies using appropriate laboratory tests or if the biting animal remains healthy for more than 10 days from the date of the bite (3).

These recommendations aim to reduce the fatal risk of clinical rabies while increasing access through judicious use of PEP vaccines which are scarce in most regions where the disease is endemic. In addition to the use of rabies biologicals, the risk of rabies is further reduced through thorough wound washing to flush as much of the virus from the bite wound as possible. Removing the constraints to access to life-saving PEP protects the health of vulnerable populations and ensures social justice (11). Epidemiological and economic modeling has shown that investment by Gavi, the Vaccine Alliance, would be an extremely cost-effective intervention reducing the burden of rabies and catalyzing mass dog vaccinations to eliminate the disease (12).

The provision of prompt and appropriate PEP to bite patients is however predicated on a healthcare workforce that is well-informed on the assessment of rabies risk, rabies prevention and management, and a healthcare system that promptly avails rabies biologicals to patients at risk of the disease. Here, we assessed the knowledge and awareness on rabies and its management among healthcare workers and the availability of rabies biologicals in Makueni County, a region in rural Kenya implementing rabies elimination activities.

## Materials and Methods

### Study Design

This study was conducted in Makueni County. Makueni County is one of the five pilot counties implementing rabies elimination activities to achieve freedom from dog-mediated human rabies by 2030 (7).

**Figure 1:**
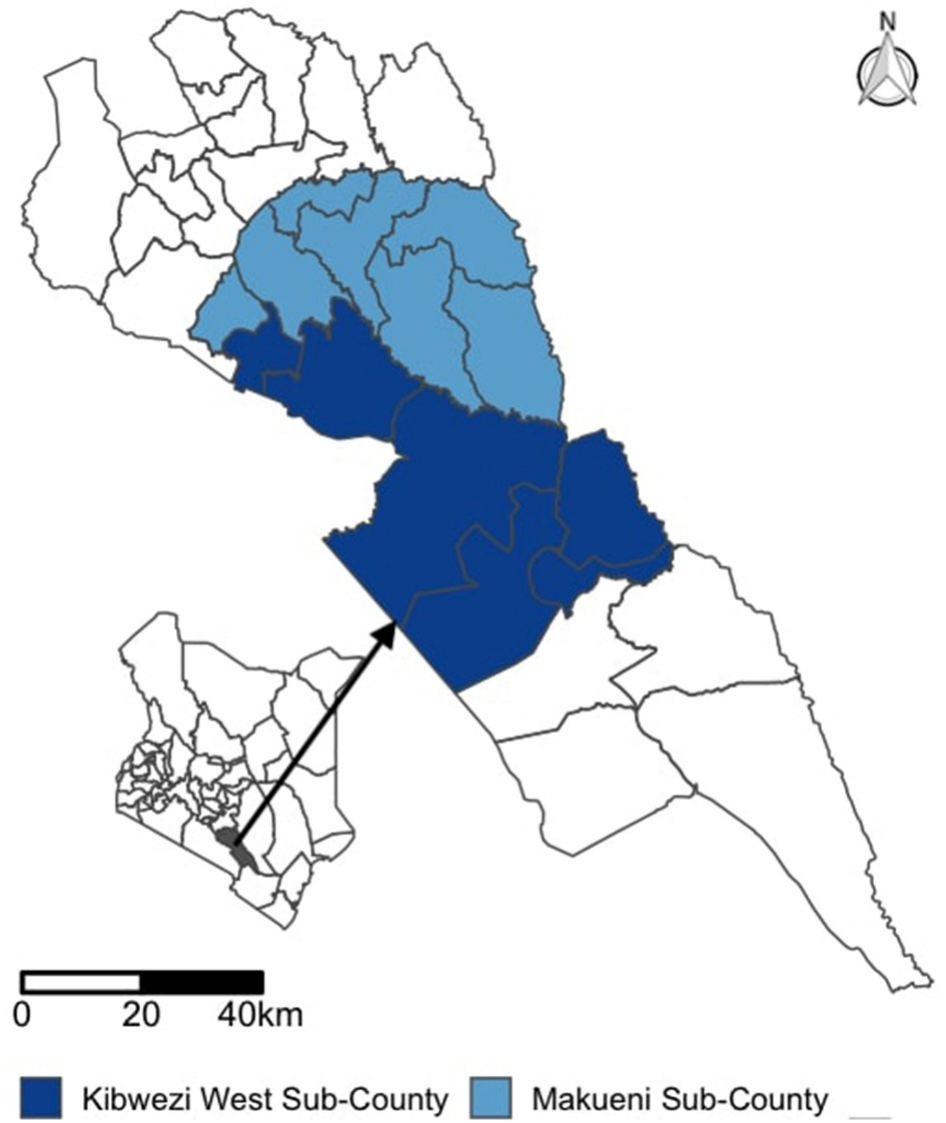
Map showing the study area comprising of sub-Counties in the South Eastern Kenya in the County of Makueni.

The County lies in the South-Eastern part of Kenya covering an area of 8,177 Km^2^ and has an estimated population size of 9,87,653 as at 2019 (13). The County is divided into six sub-counties (Mbooni, Kaiti, Makueni, Kibwezi East, Kibwezi West, and Kilome). Makueni and Kibwezi West sub-counties with a total of 13 wards and 144 healthcare units were selected as the study areas (Figure 1). The two sub-counties selected for the study had ongoing rabies surveillance programs. Makueni subcounty is the County headquarters and has the highest population of 1,93, 802 while Kibwezi West has a population of 1,65, 933. The healthcare system in Kenya is classified into four main tiers/levels of care: tier 1—community services aimed at creating demand for health services; tier 2—primary health services comprising of maternity homes, dispensaries, and health centers; tier 3—county referral hospitals that form a network of comprehensive health services run by the counties; and tier 4—the national referral services that provide highly specialized health care services including training, research and setting the standards for quality patient care (14). For this study, we included three tier 3 hospitals (the county hospital and two-sub county hospitals), and 39 tier 2 health units randomly selected from the list of all health units (20 health centers and 121 dispensaries and clinics). The selection of the 42 health facilities was dependent on the availability of funds while the number of healthcare workers interviewed was dependent on the level of the health facility, the availability of the healthcare workers and their consent to participate in the study. The study was conducted in November 2018. Rabies elimination activities started in Makueni County in 2015 with dog census, pilot vaccination campaigns, training of healthcare workers on rabies, and awareness campaigns in schools and market places. The training of healthcare workers was conducted through continuous medical education (CME) at the health facilities with several topics covered including bite wound management, rabies clinical signs and PEP administration. However, not all healthcare workers will attend all CME sessions, and healthcare workers frequently change duty stations. Starting 2016, the County has been implementing mass dog vaccination campaigns mainly by setting up static vaccination centers where dogs are brought to receive their rabies vaccination. These campaigns have resulted in vaccination coverage of dogs above 40% and varying by year and by geographical regions of the County.

### Study Participants

At each study health facility, we targeted to interview medical officers (defined as holders of Bachelor of Medicine and surgery degree), nurses (holders of a diploma or bachelor's degree in nursing), clinical officers (holders of diploma in clinical studies), pharmacists, pharmacy and laboratory technologists, and public health officers who were available and willing to participate in the study. The cadre of staff placed in each health facility is based on the level of care offered at the facility. The majority of respondents were nurses and clinical officers in the tier 2 facilities. Medical officers were only found in tier 3 facilities.

### Data Collection, Management, and Analysis

We developed a structured questionnaire to collect data from health care workers on their demographics, professional qualifications, work experience, knowledge, and awareness on rabies and its management. Data on knowledge of rabies included categorization of dog bite wounds and assessment of the risk of rabies, PEP regimens, site and route of administration, management of human rabies cases, diagnosis methods including differential diagnosis for acute encephalitis cases and sample collection, transportation, and laboratory confirmation. At the health-facility level, we collected data on the availability of anti-rabies vaccines, rabies immunoglobulins, and periods of stock-out experienced between 2018. The questionnaires used are provided in Appendix 1.

The study questionnaire was programmed in CommCare tool to allow for electronic data capture using mobile phones. The questionnaires were pre-tested before being administered to the healthcare workers. Data cleaning and analysis were conducted using the R statistical computing language (15). We used descriptive statistics to report proportions of healthcare workers with specific knowledge and awareness on rabies, and availability of rabies biologicals within health facilities.

### Ethical Clearance

The study received ethical approval from Kenya Medical Research Institute/Scientific and Ethics Review Unit (Ref No. KEMRI/SERU/CGHR/046/3268).

## Results

### Demographic Characteristics of the Respondents

In total, 73 healthcare workers from 42 health facilities consented and participated in the study. Most of the respondents were female (69%). The participants' ages ranged from 22 to 60 years and had a mean of 36 years (median 34 years). The years of work experience as healthcare workers ranged from 1 month to 38 years (median 7 years), with most (62%) having five or more years of work experience. Details of the demographics, cadre of healthcare workers, education level, years of experience, and the number of respondents for different levels of health facilities are provided in Table 1. Majority (86%, *n* = 63) of the healthcare workers reported to have learnt about rabies disease in at the college level and 12% (*n* = 9) from the work place.

**Table 1:** Socio-demographic characteristics of healthcare workers that participated in the study.

**Variable**	**Characteristic**	**Number** **(*n* = 73)**	**Percentage**
Medical qualification	Doctors	2	2.7%
	Nurses	47	64.4%
	Clinical officer	9	12.3%
	Pharmacist	2	2.7%
	Clinical Pharmacist	1	1.4%
	Laboratory technologist	9	12.3%
	Pharmacy technologist	1	1.4%
	Public health officer	2	2.7%
Education level	Master's degree	1	1%
	Bachelor's degree	8	11%
	Diploma	57	78%
	Certificate	7	10%
Work experience *n* = 68 (years)	<4	26	38%
	5–10	21	31%
	11–20	10	15%
	21–30	8	12%
	31–40	3	4%
Health facility (*n* = 42)	Tier 2	39	93%
	Tier 3	3	7%

### Dog Bites and Their Management

Forty-one (56%) of the respondents reported having seen a dog-bite patient in the one month preceding the study interview (range 1–20 dog bite patients). Only 17 (23%) of the study participants were aware of the WHO categories of bite wounds that guide the use of post-exposure vaccines following contact with suspected rabid animals. We did not find any statistical associations between age of the respondent or years of experience with participant's knowledge on the WHO bite categories. A brief description of each WHO bite category was provided to each respondent and were then requested to describe how they would manage patients under each bite category. Nine (12%) of the respondents did not know how to manage bite patients under any of the three categories.

Among the 64 healthcare workers that reported knowing how to manage bite patients, 13 (20%) reported they would administer PEP to bite category I patients, which is not recommended as per WHO guidelines. Thirty-nine (61%) and 53 (84%) respondents indicated they would administer PEP to patients with category II and III, respectively. Only three (5%) of the respondents indicated they would inject RIG for bite category III patients.

**Figure 2:**
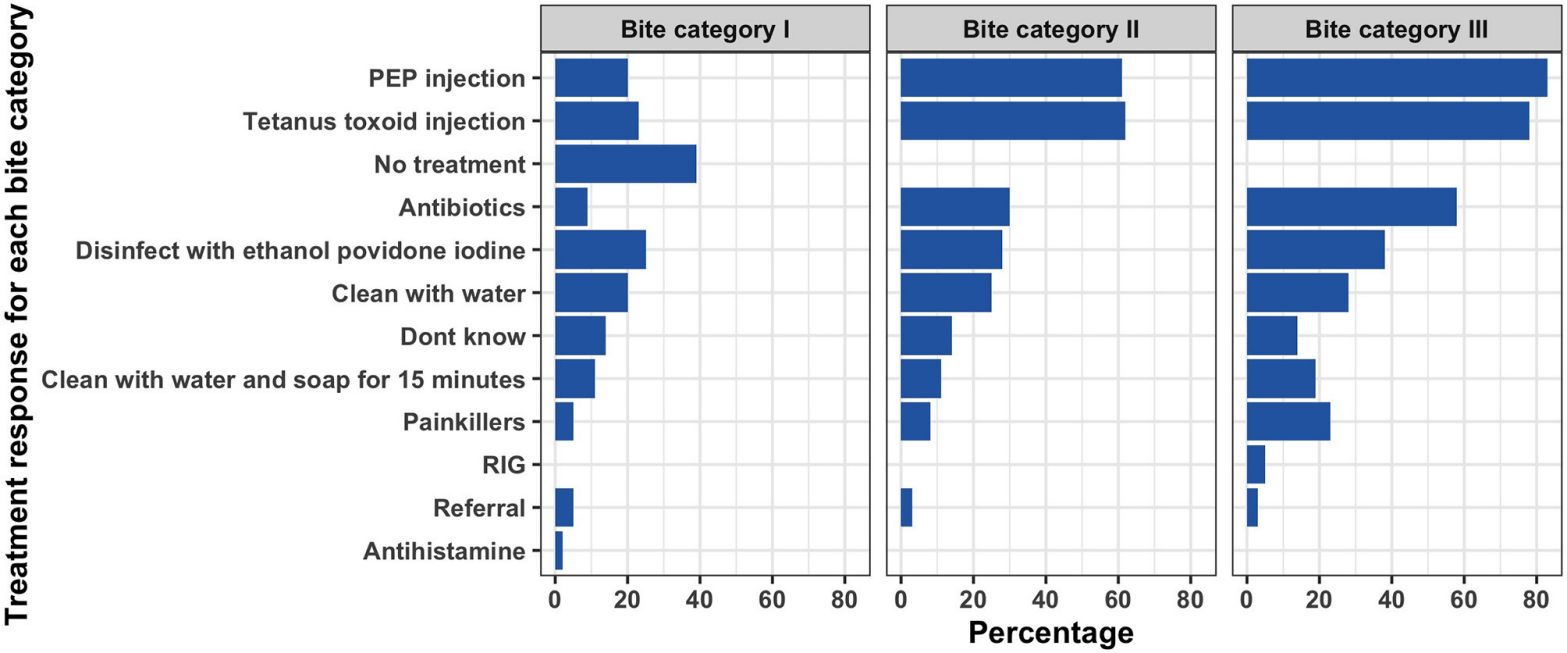
Responses by healthcare workers on how they would manage patients with bites under each of the WHO bite categories.

Thorough wound washing with running water for at least 15 min is recommended as part of the post-exposure treatment of bite wounds. Only 22 (33%) and 28 (43%) of the respondents reported they would clean the wound with water (either with water or with water and soap) for bite category II and III, respectively. Figure 2 summarizes treatment responses for each bite category.

### PEP and RIG Administration

**Figure 3:**
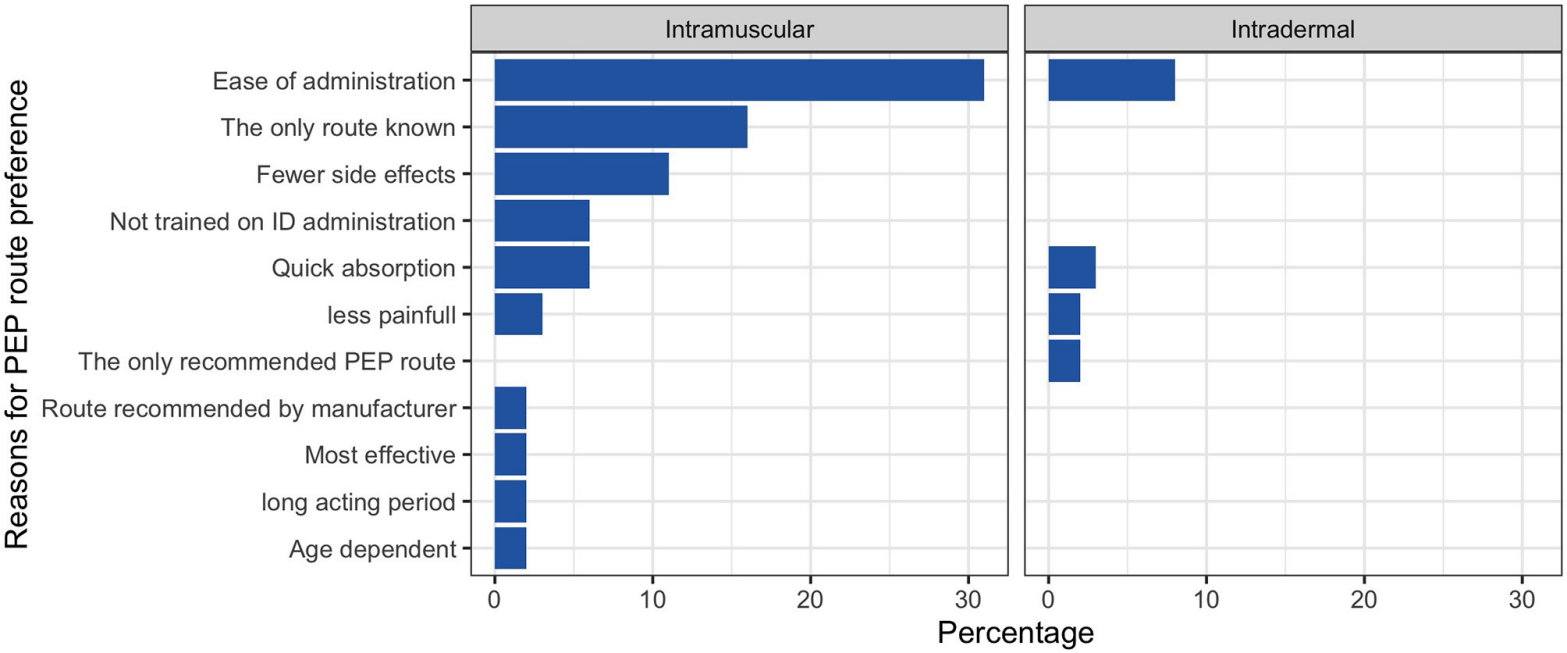
Reasons for preference of each route of rabies vaccine administration among the healthcare workers.

We sought information from the respondents on the number of PEP doses required for bite patients. Fifty-five (75%) of the respondents indicated five doses, with 8 (11%) indicating they did not know the number of PEP doses. On the route of vaccine administration, 53 (73%) of the respondents mentioned the intramuscular (IM) route, 9 (12%) did not know, 6 (8%) both IM and intradermal (ID), and 2 (3%) ID only. Only 7 (10%) of the study participants knew there were differences in the number and volume of doses when using IM and ID routes of PEP administration. IM route was the preferred route of PEP administration (70% of the respondents) compared to the ID route (15%). The rest either did not have a preference or did not know of the vaccine administration routes. Ease of administration was the most common reason among the respondents (38%) that preferred the IM route, and the deltoid muscle was the preferred site of injection for 78% of the respondents. Figure 3 provides a summary of the reasons for the preferences of IM and ID route of vaccine administration.

Fifty-four (74%) respondents could not name a brand name of PEP vaccine available in Kenya. The respondents provided information on the circumstances under which a bite patient would discontinue PEP injections with 37 (51%) reporting PEP could be discontinued when the biting animal was alive and without clinical signs after 14 days of confinement whereas 22 (31%) didn't know the circumstances under which PEP could be discontinued. Table 2 summarizes the participants responses on when PEP administration could be stopped.

**Table 2:** Responses on circumstance under which PEP should be discontinued.

**Response**	**Number (%)**
Biting animal doesn't show rabies clinical signs after 14 days of confinement	37 (51%)
Availability of biting dog vaccination certificate	9 (12%)
PEP side effects	6 (8%)
Negative laboratory result of the biting animal	5 (7%)
Don't know	22 (31%)
Patient develop clinical signs of rabies	2 (3%)
Patient bite wound healed	1 (1%)
Under no circumstance should bite patient discontinue PEP	1 (1%)

Of the 9 respondents that mentioned they would rely on the availability of biting dog vaccination certificates to inform whether to discontinue PEP, six reported they would check for validity of the biting dog vaccination certificate by checking the period between vaccination to the next booster (information on the time since the last vaccination that was safe was not captured).

When asked if they were aware of RIG, only 13 (18%) of the participants indicated awareness of RIG. Only 6 of those reported RIG to be an indication for category III bites, 4 knew of either Human Rabies Immunoglobulin (HRIG) or Equine Rabies Immunoglobulin (ERIG) and the dosages.

### Healthcare Workers' Knowledge on the Clinical Presentation of Human Rabies

The respondents were asked about the clinical signs that would make them suspect a case of human rabies. Nearly two-thirds of the respondents reported abnormal vocalization as the main clinical sign, with hypersalivation, aggressiveness, hydrophobia, and paralysis being frequently mentioned.

### Rabies as a Differential Diagnosis for Acute Encephalitis

In areas endemic for rabies, the inclusion of the disease as a differential diagnosis for cases of unexplained acute encephalitis presenting at health facilities is critical for effective rabies surveillance (3). Six (8%) of the 73 respondents reported having encountered patients with acute encephalitis (two doctors, two nurses, one clinical pharmacist and one clinical officer). However, only one of these six considered rabies as a differential diagnosis of acute encephalitis (three mentioned cerebral malaria, five cryptococcal meningitis, and one streptococcal meningitis).

### Knowledge on Management, Diagnosis, and Confirmation of Human Rabies

We obtained information from the healthcare workers on how they would manage clinical cases of human rabies. Fourteen (19%) reported they did not know how they would manage a patient, 43 (59%) reported they would isolate the patient, 14 (19%) would sedate the patient while 11 (15%) would treat symptomatically. Seven (10%) of the respondents indicated they would refer the patient to a higher-level health facility with a few indicating they would intubate the patient, provide supportive care or give PEP.

When asked how confirmation of rabies is made, only five (7%) of the respondents indicated through testing of samples collected at post-mortem (three of the respondents indicating brain samples, one reported the use of skin biopsy while one could not provide information on which sample was required for diagnostic testing). A quarter of the respondents did not know how confirmation of human rabies was done, while more than half of the respondents reported this could be done through clinical signs. Twenty-three (32%) of the respondents indicated confirmation could be done through testing of antemortem samples (16 citing saliva and serum, six cerebrospinal fluid, three skin biopsies from the nape of the neck as the samples of choice). The frequency of responses on ways of confirming rabies is provided in Table 3. Among those that indicated post-mortem or antemortem samples were required to make a confirmatory diagnosis for rabies, two-thirds reported they would package the samples in a water-tight and leakproof container, 5 (18%) would ship samples overnight frozen on dry ice while 3 (11%) would store samples at −20^o^C before shipping the samples. Six (21%) of the respondents reported not to know how they would package and transport the rabies samples.

**Table 3:** Response on how to confirm a case of human rabies.

**Response**	**Frequency**
Clinical signs	41 (56.1%)
Collection of ante mortem samples	23 (31.5%)
Don't know	19 (26%)
Collection of postmortem samples	5 (6.8%)
Patient history	2 (2.7%)

Two-thirds of the respondents indicated they were not comfortable with collecting samples from a suspected case of human rabies. When all the respondents were asked who they thought would be best suited to collect the samples to help confirm rabies, 53 (73%) of the participants indicated this should be done by laboratory technicians, 11 (15%) by pathologists, 11 (15%) by clinical officers, 7 (10%) by doctors, 4 (5%) by nurses, 2 (3%) by public health officers and 1 (1%) by veterinary officers. Thirty-six (49%) of the respondents were unaware of the laboratory that would conduct confirmatory tests for rabies in Kenya.

### Rabies Vaccine and RIG Availability at the Visited Health Facilities

None of the 42 health facilities reported to have ever stocked RIG. The County Referral hospital and the sub-county hospitals were all reported to store post-exposure vaccine. Among study health facilities, only 2 of 11 (18%) health centers, and 2 of 28 (7%) of study dispensaries and clinics stocked post-exposure vaccine. The two dispensaries were privately owned facilities. Among those facilities that stocked post-exposure vaccine, the stock-out period in the year preceding the study ranged from 0–28 weeks (median = 0). The healthcare workers reported lack of supply by the county government as the major (71%, *n* = 52) factor affecting availability of the vaccine at the facilities. Procurement at the facility level was influenced by the number of bite patients (55%, *n* = 40) and availability of funds (14%, *n* = 10).

## Discussion

This study highlights the inadequate knowledge about rabies prevention and management amongst healthcare providers in rural Kenya. We report that majority of healthcare workers were not familiar with WHO guidance on the categorization of bite exposures, management of dog bites, and human rabies cases, and had inadequate knowledge on the use of dose-sparing vaccination regimens and indications for administration protocols for RIG. Moreover, healthcare workers were unlikely to consider rabies as a cause of encephalitis and the supply of rabies biologicals was inadequate. These results point out the need to both educate healthcare workers on rabies and improve the provision of PEP at health facilities. Mass vaccination of dogs and PEP are two main strategies that have worked in ending deaths from rabies in many regions of the world and are key strategies in the global goal for eliminating human rabies death set for 2030 (5, 16–19). To effectively deliver these two interventions, community engagement (that creates awareness of rabies leading to a reduction in rabies exposures, participation of dog owners in dog rabies vaccination campaigns, and appropriate first-aid and health-seeking following bites) and appropriate knowledge among healthcare workers, as well as availability of and accessibility of rabies biologicals for bite victims (20–22).

Here we found inadequate awareness of WHO categorization of rabies exposure and appropriate PEP provisioning suggesting sub-optimal management of bite patients. Recent studies have highlighted the challenges in PEP availability and access in many low-and-middle-income countries (8, 23). In such settings, risk assessment is important to ensure judicious use of PEP when available. In this study, a section of healthcare workers reported PEP was indicated for Category I exposures. The WHO guidelines on the use of PEP only indicate the use of PEP for bite categories II and III (3). Understanding the use of PEP and patient risk assessment is important to prevent unnecessary PEP use. Risk assessment to improve judicious use of PEP are now advocated to be part of rabies elimination programs, mainly through the integrated bite case management (IBCM) approaches that promote information sharing between the healthcare workers and their colleagues in the veterinary sector (24–27).

While the majority of study participants were conversant with the IM route of vaccine administration and some PEP regimens, there was a lack of awareness on ID administration and PEP regimens. Although the use of ID is cost-saving, dose-sparing, and likely to mitigate vaccine shortages in rabies endemic countries and is now recommended by WHO (4, 10), most of these countries are predominantly using the IM route (8, 23, 28). The low awareness of the use of ID for PEP suggests this as an important educational topic for supporting rabies elimination activities. ID use is most beneficial in health facilities that receive multiple bite patients within 24 h periods as one vial may be used to treat several patients, and has the potential to significantly increase availability and accessibility of PEP. This however does not mean ID use in single daily case health facilities is disadvantageous or wasteful compared to IM use as the same number of patients are prevented from clinical rabies even when the remaining ID doses are discarded. ID use is however more technical than IM administration and requires additional training of healthcare workers to effectively administer the vaccine. RIG is recommended in combination with PEP for category III bite exposures where the patients have severe bites to the head, face, and neck to provide passive immunity through the delivery of virus neutralizing antibodies at the site of the bite before vaccine-induced virus-neutralizing antibodies can take effect (4). RIG was not available in any of the study facilities, and only a few healthcare workers knew of RIG, types, and dosage. Similar unavailability of RIG in health facilities has been reported in other studies in Kenya and other rabies endemic countries (8, 23, 28). For rabies elimination programs, the most critical components of PEP are thorough wound washing coupled with immediate administration of rabies vaccines and completion of the PEP course, which have been shown to prevent rabies in >99% of bite patients (29). The unavailability of PEP at the health facilities may contribute to low awareness of rabies if the healthcare workers rarely use it during provision of treatment and care.

Although most of the respondents could identify the clinical signs associated with human rabies, there was limited awareness and knowledge on the appropriate ante and post-mortem samples required for rabies diagnosis, who should collect them, or where rabies diagnostic tests were carried out in Kenya. This low awareness and knowledge among healthcare workers may hinder the effectiveness of rabies surveillance in supporting the goal of elimination and for monitoring and assessing control measures, including verification of rabies elimination, and maintenance of rabies freedom post-elimination (30). Obtaining a proper history of a dog bite or exposure is important in many rabies endemic settings, where confirmation of rabies after bites is rare. Previous studies have shown how easily rabies is missed especially where diseases such as cerebral malaria that result in acute encephalitis, a good differential diagnosis for rabies, are common (31).

COVID-19 pandemic has disrupted many essential health services including strategies for rabies elimination (32) with funding for non-COVID-19 disease projects redirected to control of COVID-19. These has disrupted mass dog vaccination, provision of PEP and community awareness activities (33). Funding rabies elimination programs will require local ownership and financing from governments in endemic countries such as Kenya. Elimination success is however likely with greater investments besides domestic financing, as has been demonstrated for successful programs such as smallpox and rinderpest eradication, polio eradication, malaria elimination, among others. Rabies elimination in the dog population is the cost-effective strategy for eliminating cases in humans. Arguably, funding including from GAVI should include support for mass dog vaccinations if the elimination activities are to be cost-effective.

Although this study revealed notable low awareness and knowledge on rabies and its management among healthcare workers, it had a few limitations. The first was that relatively few healthcare workers were available and consented to participate in the study. The result was that certain cadres of healthcare workers such as doctors (who are only found in the higher-level health facilities) were only represented by a few individuals. Bite patients will however likely report to the lowest healthcare units and have their first contact with lower-level facility healthcare workers such as nurses. Although some of the facilities where data was collected had representatives receiving awareness on rabies and training on dog bite management, the facilities has high healthcare workers turnover where those trained could have been transferred to other departments in the facilities or to other health facilities which could result to low awareness. Although the study was conducted in south-eastern Kenya which is identified as a rabies hotspot (7), the findings may not necessarily be an adequate representation of the healthcare workers' knowledge and awareness on rabies elsewhere in the country. During the study, the healthcare workers did not have a chance to consult medical reference materials or colleagues, which may be also a common practice among healthcare workers when faced with bite patients or suspect rabies cases. While the awareness and knowledge on rabies is low, it may still be an overestimate among general healthcare workers population since the study was conducted in an area currently implementing rabies elimination activities where some healthcare workers were sensitized on rabies. This study did not gather any data on confirmed cases of rabies or rabies related deaths. Confirmation of rabies cases is through testing of samples collected during postmortem examinations—which are seldom conducted in the country, as is in many countries where rabies is endemic. From the data, we found a low suspicion index for rabies cases, which may compound the surveillance and confirmation cases of rabies in the region.

## Conclusion

Rabies continues to pose a significant public health concern in Kenya. Prevention of clinical cases of human rabies following exposure is dependent on prompt access to appropriate PEP. Improving access to rabies PEP, by making post-exposure vaccines available and free at the point-of-care in disease endemic areas is now seen as a feasible and highly cost-effective strategy to contribute to ending rabies deaths, and a socially just way to redress the inequity associated with rabies (11, 12). High levels of awareness and knowledge on rabies by healthcare workers is critical in supporting rabies elimination programmes. Our study highlights opportunities to tailor healthcare training programs for rabies elimination and emphasis on rabies prevention and control in preservice training, as well as part of continuous medical education. Appropriate risk assessment of dog bites to support judicious use of PEP is required. This is supported by the recommendation of establishing integrated bite case management that promote information sharing between the healthcare workers and the veterinary sector (12). Proper management of humans exposed to the rabies virus remains a critical step toward achieving zero human deaths from rabies by 2030. Integrating the provision of PEP, adopting the latest WHO recommendations for PEP including the use of intradermal route of vaccine administration, risk assessment through sharing information between the health and veterinary sectors, and continuously training healthcare workers on proper management of bite patients and human rabies cases including diagnosis, are all critical for the elimination of rabies in Kenya and reaching the zero human death from rabies by 2030.

## Data Availability Statement

The dataset generated and analyzed for this study is available from Open Science Framework: https://osf.io/a6n93/. This dataset is available under a CC0 1.0 Universal license.

## Ethics Statement

The studies involving human participants were reviewed and approved by Scientific and Ethics Review Unit - Kenya Medical Research Institute. Written informed consent for participation was not required for this study in accordance with the national legislation and the institutional requirements.

## Author Contributions

VC: conceptualization, data curation, formal analysis, methodology, supervision, writing—original draft preparation, and writing—review and editing. PK: supervision and writing—review and editing. PB: methodology and writing—review and editing. DK, MMut, AM, CN, MMar, and TB: writing—review and editing. NM: data curation, formal analysis, and writing—review and editing. TB: writing—review and editing. SD: resources and writing—review and editing. KH: conceptualization, formal analysis, funding acquisition, methodology, resources, and writing—review and editing. ST: conceptualization, funding acquisition, project administration, resources, supervision, and writing—review and editing. All authors contributed to the article and approved the submitted version.

## Funding

ST and KH had funding support from the Wellcome Trust (Grant numbers 110330/Z/15/Z and 207569/Z/17/Z respectively). This study was partly funded through a grant from Sanofi to Kenya Medical Research Institute. VC receives funding support from the Fogarty International Center and the Institute of Allergy and Infectious Diseases of the National Institutes of Health under Award Number D43TW011519.

## Author Disclaimer

The content is solely the responsibility of the authors and does not necessarily represent the official views of the National Institutes of Health.

## Conflict of Interest

SD is an employee with Sanofi and may hold shares and/or stock options in the company. The remaining authors declare that the research was conducted in the absence of any commercial or financial relationships that could be construed as a potential conflict of interest.

## Publisher's Note

All claims expressed in this article are solely those of the authors and do not necessarily represent those of their affiliated organizations, or those of the publisher, the editors and the reviewers. Any product that may be evaluated in this article, or claim that may be made by its manufacturer, is not guaranteed or endorsed by the publisher.
