# Supplementary material for: Rabies Elimination in Rural Kenya: Need for Improved Availability of Human Vaccines, Awareness and Knowledge on Rabies and Its Management Among Healthcare Workers
Source: Front Public Health. 2022 Mar 10;10:769898. doi: 10.3389/fpubh.2022.769898 (PMC8960031; doi:10.3389/fpubh.2022.769898)
Supplement: Supplementary file 1 [file Data_Sheet_1.docx]

**APPENDIX 1: A QUESTIONNAIRE ON ASSESSING RABIES AWARENESS AND BITE PATIENT MANAGEMENT AMONG HEALTHCARE WORKERS IN MAKUENI COUNTY**

**SECTION 1: IDENTIFICATION**

| **HEALTH FACILITY** |  | |
| --- | --- | --- |
| **LEVEL OF THE HEALTH FACILITY** |  | |
| **INTERVIEWER ID** |  | |
| **INTERVIEW DATE** |  | |
| **LOCATION** | **SUBCOUNTY** |  |
|  | **WARD**  (insert GPS coordinates) |  |

**SECTION 2: SOCIO-DEMOGRAPHIC CHARACTERISTICS**

| No | Questions | Coding categories |
| --- | --- | --- |
| 201 | Sex | Male:  Female: |
| 202 | How old are you? |  |
| 203 | Profession | 1. Doctor 2. Nurse 3. Clinical officer 4. Pharmacist 5. Clinical pharmacist 6. Laboratory technologist 7. Pharmacy technologist 8. Others (specify) |
| 204 | When did you start working? |  |
| 205 | Highest level of education completed | 1. Certificate 2. Diploma 3. Bachelors 4. Masters 5. Phd 6. Others (specify) |

**SECTION 3: KNOWLEDGE**

| 301a | Has a case of acute encephalitis presented in the health facility in the last one year? | 1. Yes 2. No 3. Don’t know |
| --- | --- | --- |
| 301b | If yes, what were your differential diagnoses? List them in order of importance | 1. Rabies 2. Streptococcal meningitis 3. Cryptococcal meningitis 4. CMV virus 5. Cerebral malaria 6. Others |
| 302 | Do you know WHO categorization of dog bite wound? | 1. Yes 2. No |
| 303a | How many bite wound categories are there? | 1. 1 2. 2 3. 3 4. Others |
| 303 | What are the distinguishing features of dog bite wound? | Check box   1. Touch but skin is intact 2. Scratch/abrasion, no bleeding 3. Single/multiple bites/scratches, licks on broken skin, contamination of mucus membrane with saliva from licks. 4. I don’t know 5. Others (specify) |
| 304a | Have you come across a human dog bite case in the last 1 month? | 1. Yes 2. No |
| 304b | If yes, how many? |  |
| 305a | How would you manage a patient that presents with a dog bite wound? | 1. Clean bite wound with water 2. Clean bite wound with water and soap for 15 minutes 3. Disinfect bite wound with ethanol/ povidone iodine 4. Tetanus toxoid injection 5. Give antibiotics 6. Anti-rabies vaccine injection(PEP) 7. Administration of RIG 8. Painkillers 9. Debridement of devitalized tissue 10. Closing up the wound 11. Non wound suturing 12. Don’t know 13. Others (specify) |
| 305b | For those that mentions RIG, what is the indication for administering RIG? |  |
| 305c | How is RIG administered/applied? | 1. Infiltration into the bite wound 2. Muscle injection 3. Don’t know 4. Others (specify) |
| 305d | What is the dosage for RIG? | 1. ERIG 40IU/KG 2. HRIG 20IU/KG 3. Don’t know |
| 306a | For those who do not mention RIG, have you heard about RIG? | 1. Yes 2. No |
| 306b | If yes, to whom is it indicated to? |  |
| 306c | How is it administered/applied? | 1. Infiltration into the bite wound 2. Muscle injection 3. Don’t know 4. Others (specify) |
| 306d | What is the dosage for RIG? | 1. ERIG 40IU/KG 2. HRIG 20IU/KG 3. Don’t know |
| 307 | In case of a bite, how many doses of anti-rabies vaccine does a patient require? | 1. I 2. II 3. III 4. IV 5. V 6. Don’t know 7. Others (specify) |
| 308 | What is the regimen of rabies post exposure prophylaxis vaccine? | Check box   1. 0-3-7-14-28 (IM) 2. 0-3-7 (ID)???? 3. Both 1 and 2 4. Don’t know 5. Others (specify) |
| 309 | What would be the dates of injection if a dog bite patient presented to the health facility on 1^st^ May? | 1. 1^st^-4^th^-8^th^-15^th^-29^th^ 2. Don’t know 3. Others (specify)   By asking this question, I want to find out if they know how to count the different days |
| 310 | What is the route of administration for anti-rabies vaccine? | 1. Intramuscular 2. Intradermal 3. Both 1 and 2 4. Don’t know 5. Others (specify) |
| 312 | What is the difference between intramuscular and intradermal route of administration for anti-rabies vaccine? | 1. Number of doses 2. Volume of each dose 3. Site of administration 4. Don’t know 5. Others |
| 313 | Which route do you prefer? Why? |  |
| 314 | What is the site of administration for anti-rabies vaccine? | 1. Deltoid muscle 2. Thigh 3. Both 1 and 2 4. Don’t know 5. Others (specify) |
| 315 | Which anti-rabies vaccines are available? | 1. Verorab 2. Rabipur 3. Immovax 4. Don’t know 5. Others (specify) |
| 316 | Under what circumstance would you advice a bite patient to stop taking anti-rabies vaccine? | 1. Laboratory results for biting animal test negative 2. Confined animal doesn’t show rabies clinical signs in 14 days 3. Whenever they feel like 4. When Pep is not available 5. When the bite wound has healed 6. others |
| 317a | Have you ever come across a rabies suspect patient in the last 5 years? | 1. Yes 2. No 3. Don’t know |
| 317b | If yes, how many cases? | 1. 1-5 2. >5 |
| 317c | When? | In years |
| 327d | How many cases have you seen in the last 1 year? | 1. 1-4 2. 5 and above |
| 317e | What were the reasons for you suspecting rabies? | Check box   1. Visible dog bite wound 2. History of dog bite 3. Patient presented with rabies clinical signs 4. Patient negative for bacterial, parasitic, viral, fungal and non-infectious meningitis 5. Patient negative for cerebral malaria 6. Others(specify) |
| 317f | What were the clinical signs |  |
| 318 | What are the clinical signs that would make you suspect human rabies? | Check box   1. Hydrophobia 2. Aerophobia 3. Aggressiveness 4. Excessive salivation 5. Abnormal vocalization 6. Pain at site of bite 7. Fever 8. Nausea 9. Paralysis 10. Hypersexuality 11. Presence of bite wound 12. Other (specify) |
| 319 | Do you know of any human bite case that died of rabies after receiving full course of PEP? | 1. Yes 2. No |
| 320 | How would you manage a confirmed case of human rabies? Check all that apply | 1. Isolate 2. Sedate 3. Treat symptomatically 4. Intubation 5. Do nothing 6. Don’t know 7. Others (specify) |
| 321 | How would you confirm a case of human rabies? | 1. Collect ante-mortem samples 2. Collect post-mortem samples 3. Both 1 and 2 4. Use clinical signs 5. Not possible 6. Don’t know 7. Others (specify) |
| 322 | For those who mention ante-mortem samples, What ante-mortem samples would you provide for rabies diagnosis? | 1. Saliva 2. Skin biopsy at the nape of the neck 3. CSF 4. Serum 5. Don’t know 6. Others (specify) |
| 323 | What post-mortem samples would you provide for rabies diagnosis? | 1. Brain sample 2. Skin biopsy 3. Don’t know 4. Others(specify) |
| 324 | How would you package, store and transport samples collected for rabies diagnosis? | Check box   1. Watertight and leakproof container 2. Store at -20^o^C 3. Ship samples frozen on dry ice overnight 4. No additives/fresh samples 5. Don’t know 6. Not applicable 7. Others (specify) |
| 325 | Who would/should collect human samples for rabies diagnosis? | 1. Doctor 2. Nurse 3. Clinical officer 4. Pathologist 5. All 6. Don’t know 7. Others (specify) |
| 326 | Would you be comfortable collecting human samples for rabies diagnosis? | 1. Yes 2. No 3. Don’t know |
| 327a | Do you know of any laboratory that would carry out human ante-mortem and post-mortem rabies test? | 1. Yes 2. No |
| 327b | If yes, which one? |  |
| 328 | Does your health facility have RIG? | 1. Yes 2. No |
| 329 | Does your health facility have anti-rabies vaccine? | 1. Yes 2. No 3. Don’t know |
| 330 | What is the brand of PEP available at your health facility? |  |
| 331a | In the last 1 year, how many weeks has your health facility gone without PEP? |  |
| 331b | What are the factors affecting PEP availability at your health facility? | 1. Lack of funds 2. Lack of cold chain system 3. No bite patients 4. Others (specify) |
| 331c | What factors influences procurement of PEP at your health facility? | 1. Availability of funds 2. Number of bite patients 3. Seasonality 4. Nothing 5. Others (specify) |
| 332 | How are suspected/confirmed cases reported? |  |
| 333 | What do you think is the number of people dying of rabies in your County? |  |
| 334 | What do you think is the number of people dying of rabies in the Country? |  |
| 335 | What do you think should be done in order to prevent rabies? |  |
| 336 | Do you know any effort by the County to eliminate rabies? | 1. Yes 2. No |
| 337 | How much are patients charged for PEP at your health facility? |  |
| 338 | Where did you learn about rabies? | 1. School 2. Work place 3. Friend 4. Internet 5. Rabies surveillance project |
